# Adrenalin Chloride and Hydrochlorate of Cocaine in Dentistry

**Published:** 1903-10

**Authors:** R. Menger

**Affiliations:** San Antonio, Texas


### TEXAS MEDICAL JOURNAL.

ESTABLISHED JULY, 1885.

PUBLISHED MONTHLY.—SUBSCRIPTION $1.00 A YEAR.

Vol. XIX. AUSTIN, OCTOBER, 1903. No. 4.

### Original Contributions.

For Texas Medical Journal.

### Adrenalin Chloride and Hydrochlorate of Cocaine in Dentistry.

BY R. MENGER, M. D., SAN ANTONIO, TEXAS.

In the latest "Review on Adrenalin Therapy," by Parke, Davis & Co., I notice, under heading "Anæsthesia," that the absorption of cocaine and the subsequent alarming systemic effects of that drug can be prevented by a preliminary application of solution adrenalin chloride to the parts to be anæsthetized—dependent upon the reduction in caliber of the blood vessels, whose capacity for absorption is thus reduced to a minimum—the local effects of the cocaine upon the peripheral nerve-ends not being interfered with. (P. L. Anderson, M. D., New York City). Also Dr. M. A. Goldstein, St. Louis, finds that solution of adrenalin chloride very materially reduces the toxic effect of cocaine and prevents the collapse frequently following its application to the nose. * * * It is his practice to apply a 5 per cent solution of cocaine to the field of operation and to follow within a few minutes with the 1:1000 solution of adrenalin chloride. The author is of the opinion that the rather enforced contraction of the capillaries, provoked by the adrenalin solution, prevents the too liberal absorption of the cocaine.

In my late article in the Texas Medical Journal, I suggested, relying on the well-known physiological action of adrenalin chloride, to use same in bites from venomous reptiles and insects—in

2 Mj conjunction with other neutralizing remedies. Now, besides the above preliminary remarks on adrenalin chloride as a general hemostatic, etc., I have had occasion this date, August 3rd, to test the combined action of adrenalin and cocaine in a case of dentistry —extraction of twelve teeth at one sitting. The effect was so wonderful that I will give a closer account, in as much as I am not aware of any data that these two remedies combined ever have been used before in dentistry. At first I was inclined to have the dentist, Dr. Collier, of this city, use the adrenalin locally by merely rubbing a 1:1000 adrenalin solution along the gums and using the combined solution afterward, but I concluded to have both solutions used at once—a 5 per cent cocaine solution and one-half part of a 1:1000 adrenalin solution—the dentist injecting several drops with a dental syringe along the alveoli. The first attempt was made with an injection at the outer and inner side of the last lower molars, where also an old broken-off root had to be extracted. About two minutes after the injection, the dentist extracted the roots and a decayed molar—without the least pain—the only slight pain being experienced whilst the needle was introduced, but the subsequent extractions were all painless after the injection, and with but very little oozing of blood.

After these two were extracted, the solution was again injected along the other four teeth, and, it was interesting to note that when the fifth tooth was attempted to be extracted it could be done, although quite painful, but the sixth tooth was so painful that the injection had to be continued on this one and all the balance, which were then extracted perfectly painless and with but very slight hemorrhage.

The immediate effects and after-effects on above patient (a lady) were decidedly more effective and pleasant than under chloroform narcosis. The patient already had been subject to neurasthenia and its sequelæ, consequent to bad teeth, and from previous experience in having teeth extracted she is perfectly delighted over the new process. Headache, syncope, vomiting and the general *malaise* after chloroform narcosis, were absolutely absent during and after the operation, and she could have stood the extraction of a number more of teeth, or, as she said, "the entire upper row, if need be."

As stated, I believe there is no literature on the subject of using adrenalin and cocaine in dentistry, and this case may encourage others in cases in which chloroform can not well be administered. Proper precaution, of course, has to be used; the patient's general condition, pulse and respiration constantly observed, and some stimulant — cognac, etc. — given before the injections and repeated when advisable. Adrenalin itself is a powerful vasomotor and heart stimulant, and, by restricting the capillary circulation near the site of operation the other remedy is less absorbed; without that it loses its anæsthetic properties on the nerve-ends.

My report in above case, it is to be hoped, will aid the dental and medical fraternity, especially in cases where other anæsthetics or stypticis are contra-indicated. As stated, my patient had absolutely no bad after-effects—besides the main point of painless and nearly bloodless extraction of a large number of teeth at one sitting for the purpose of getting an artificial tooth set. She had bad teeth for years, with all the sequelæ of serious nervous and gastric complications, which now already are relieved to a great extent.

I had both solutions previously prepared fresh by a local druggist, and the dentist, although familiar with adrenalin, at first hesitated to use it as prepared, but afterward was at once convinced. The use of cocaine alone is considered very hazardous in extracting more than one or three teeth, but, as seen in this case, there is no risk in extracting the entire row by using both remedies combined. The patient was exceedingly nervous before we went to the dental office, therefore gave her a sedative (pas. avena) and a little cognac, and after the first tooth and roots were extracted she was so surprised (relating former experience) that the dentist proceeded with extracting the balance without the least pain and nearly bloodless. She had absolutely no bad after-effects at any time.

